# Development and calibration of an item bank for the assessment of activities of daily living in cardiovascular patients using Rasch analysis

**DOI:** 10.1186/1477-7525-11-133

**Published:** 2013-08-02

**Authors:** Harald Baumeister, Birgit Abberger, Anne Haschke, Maren Boecker, Juergen Bengel, Markus Wirtz

**Affiliations:** 1 Department of Rehabilitation Psychology and Psychotherapy, Institute of Psychology, University of Freiburg, Engelbergerstraße 41, Freiburg D-79085, Germany; 2 Medical Psychology and Medical Sociology, Medical Faculty, University of Freiburg, Freiburg, Germany; 3 Institute of Medical Psychology and Medical Sociology, University Hospital of RWTH Aachen, Aachen, Germany; 4 Department of Research Methods, Institute of Psychology, University of Education Freiburg, Freiburg, Germany

**Keywords:** Activities of daily living, Cardiovascular disease, Computerized adaptive test, Item bank, Item response theory, Rasch model

## Abstract

**Background:**

To develop and calibrate the activities of daily living item bank (ADLib-cardio) as a prerequisite for a Computer-adaptive test (CAT) for the assessment of ADL in patients with cardiovascular diseases (CVD).

**Methods:**

After pre-testing for relevance and comprehension a pool of 181 ADL items were answered on a five-point Likert scale by 720 CVD patients, who were recruited in fourteen German cardiac rehabilitation centers. To verify that the relationship between the items is due to one factor, a confirmatory factor analysis (CFA) was conducted. A Mokken analysis was computed to examine the double monotonicity (i.e. every item generates an equivalent order of person traits, and every person generates an equivalent order of item difficulties). Finally, a Rasch analysis based on the partial credit model was conducted to test for unidimensionality and to calibrate the item bank.

**Results:**

Results of CFA and Mokken analysis confirmed a one factor structure and double monotonicity. In Rasch analysis, merging response categories and removing items with misfit, differential item functioning or local response dependency reduced the ADLib-cardio to 33 items. The ADLib-cardio fitted to the Rasch model with a nonsignificant item-trait interaction (chi-square=105.42, df=99; p=0.31). Person-separation reliability was 0.81 and unidimensionality could be verified.

**Conclusions:**

The ADLib-cardio is the first calibrated, unidimensional item bank that allows for the assessment of ADL in rehabilitation patients with CVD. As such, it provides the basis for the development of a CAT for the assessment of ADL in patients with cardiovascular diseases. Calibrating the ADLib-cardio in other than rehabilitation cardiovascular patient settings would further increase its generalizability.

## Introduction

Life years with disability are becoming more prevalent in patients with cardiovascular diseases (CVD) due to higher survival rates in CVD patients and an overall increased life expectancy [1]. This development will further shift health care efforts from curative to rehabilitative interventions with a focus on patients´ functional health [2]. To assess patients´ functional status, patient reported outcomes became ever more important over the last years [3-5]. Thereby, activities of daily living (ADL) respectively physical functioning have been core measures to assess patients´ functional status [3,4,6,7].

Most often, ADL has been subdivided into basic ADL as the basic capacity to care for oneself, and instrumental ADL in reference to more complex ADL [8]. The construct “physical functioning” has been shown to consist of both basic and instrumental ADL [7]. To cover the whole spectrum of ADL, assessment instruments preferably comprise a wide range of basic and instrumental ADL [8]. Present ADL measures, however, are restricted in their breadth, and, more importantly, fail to prove unidimensionality and other psychometrical prerequisites for a valid and reliable assessment of ADL [4,9,10]. Furthermore, these assessment instruments are based on classical test theory (CTT), which has several limitations such as its focus on test scores rather than item scores and its sample dependency of item statistics [11].

Models of the item response theory (IRT) such as the Rasch model have been suggested as promising alternatives for developing questionnaires [11-13] and computer adaptive tests (CAT) [14,15]. A CAT constructs an individually tailored test for each person by means of a validated computer algorithm, developed and optimized by empirical construct and data information, with a 50% to 90% item reduction compared to paper-pencil tests [16]. By means of a computer algorithm, test items are selected on the basis of the responses to previous items, allowing for the abandonment of non-informative items [15]. As a requirement for CAT, calibrated, unidimensional item banks are needed [15]. An item bank is unidimensional if the responses to all items are determined by one construct (e.g. ADL) and the item bank is calibrated if these items have been assigned to a difficulty level (i.e. position on the latent trait). To determine the precision of this estimate, a standard error can be calculated [11,17].

To our knowledge, there are only few IRT-based ADL respectively physical functioning item banks [18-22] of which none focus on CVD patients. The calibration of the patient-reported outcomes measurement information system (PROMIS) physical function scale demonstrated substantial differential item functioning (DIF) with regard to subgroups of patients with osteoarthritis and rheumatoid arthritis [19]. It is also likely that further DIF is present for other disease groups [18,19], as patients with different forms of physical disabilities struggle with different aspects of their daily live. Thus, the aforementioned item banks might not be test-fair for CVD patients which restricts their utility in this population.

To overcome the lack of a test-fair, calibrated item bank for CVD patients, the present study aimed at the development and calibration of an item bank for the assessment of activities of daily living in cardiovascular patients (ADLib-cardio).

## Methods

### Sample and data collection

Development and calibration of the item bank for the assessment of ADL was part of the project “Development and validation of a computer adaptive test (CAT) for cardiac patients undergoing rehabilitation: RehaCAT-Cardio” [23-25]. The aim of this project was to develop and validate a CAT for cardiovascular patients with the domains “Depression”, “Anxiety”, “Activities of daily living” and “Work capacity”.

The recruitment took place between September 2009 and March 2010. A sample of 720 CVD patients was recruited in fourteen German cardiac rehabilitation centers. Clinical staff organized distribution of the questionnaires. Response rate was 35%. We included patients with essential primary hypertension (ICD-10: I10), ischemic heart disease (ICD-10: I20-25) or other forms of heart disease (ICD-10: I30-52). Exclusion criteria were inadequate German language skills, dementia or acute intoxication. All participants took part voluntarily without payment and gave written informed consent. The study has been approved by the ethic commission of the German Psychological Association.

## Materials

First, an initial pool of 349 items was developed based on (a) the Aachen ADL-item bank [26] for neurological patients and (b) an extensive literature search on ADL including 26 ADL questionnaires used in rehabilitation settings. Item identification was aimed to fit the content definitions given in the ICF [27] of the domains ‘mobility’, ‘self-supply’ and ‘domestic life’. After having translated all items into German, we excluded items due to equivalent content and lack of relevance for the assessment of ADL in patients with CVD as well as items considering cognitive functioning. Items considering basic and instrumental ADL were included. In addition, item formulations were adapted by a uniform introduction (“At the moment I’m able to accomplish the following activities without help…”) and to fit the unified consistent five-point Likert scale response format (0 (without difficulties) – 1 (with little difficulties) – 2 (with some difficulties) – 3 (with big difficulties) – 4 (impossible)). As time interval, current ability has been selected.

Second, the item pool was tested for relevance and comprehensiveness by 26 psychologists and psychocardiologists (practitioners and researchers) and 25 patients. As a result, items (a) were eliminated due to irrelevance, redundancy and incomprehensibility and (b) revised to enhance content validity and comprehensibility of the test items. Furthermore, 12 items were added to complete relevant subcategories and extreme impairment levels. Finally, the remaining item pool of 181 items (mobility: 107 items; self-supply: 31 items; domestic life: 43 items) was presented by a paper-pencil procedure to the participants of the study in two different ways. 128 patients answered all of the 181 items and 592 patients answered 20% of the items as part of a block test design. With regard to this block test design, the item pool was divided randomly into ten blocks and each participant received two blocks of items. This procedure assures missing data completely at random and allows for an unbiased large scale testing without causing extensive expenditure of time for test completion [28]. All 181 items are available on request from the corresponding author.

Socio-demographic (gender, age, family status, educational level, monthly income and employment status) and disease-specific variables (intensity of pain and subjective limitations due to CVD) were assessed by patients’ self-report. Additionally, information on specific cardiovascular diagnoses as well as comorbid mental disorders and somatic diseases was extracted from medical records.

### Data analysis

#### ***Confirmatory factor analysis***

To verify that the relationship between the items is caused by a singular factor, a confirmatory factor analysis (CFA) was conducted using MPlus [29]. Given the skewed categorical data and the root mean square error of approximation (RMSEA) as testing parameter, we used unweighted least squares (ULS) as estimator. For this pre-testing of unidimensionality, an RMSEA<0.10 can be regarded as acceptable [24,30,31].

#### ***Mokken analysis***

A Mokken analysis was computed with the program STATA [32] to examine the double monotonicity as a prerequisite of the partial credit Rasch model. Double monotonicity means that every item generates an equivalent order of person traits, and every person generates an equivalent order of item difficulties. The coefficient H of Loevinger (0.30≤H<0.40: weak scale; 0.40≤H<.50: medium scale; 0.50≤H: strong scale) was used as testing parameter [33].

#### ***Rasch analysis***

The Rasch analysis based on the partial credit model (PCM, [34]) was calculated using the program RUMM2030 [35]. Given a significant likelihood ratio test (p<0.001), the partial credit model was preferred to the rating scale model. The PCM allows for different response categories across items and evaluating monotonous ordering of category thresholds. The Rasch analysis process was based on Tennant and Conaghan [36] and is summarized below with the main procedures and critical values of the chosen parameters.

Threshold ordering: A threshold is the probabilistic turning point between two response categories, for example, the point where the probability of response category “2” gets larger than the probability of response category “1”. If categories were disordered, adjacent categories were merged.

Fit to the model: As overall fit statistic, the item-trait interaction score was used. This score reflects the hierarchical order of items across the trait. A statistically nonsignificant probability value (p>0.05; chi-square) of the item trait interaction score indicates model fit. Additionally, the statistics of the residuals for items and persons were used. A perfect model fit would be reflected by residuals with a mean of 0.00 and a SD of 1.00. Individual item misfit was determined by item fit residual values (residuals>±2.50) and item chi-square values (chi-square probabilities<0.05, Bonferroni adjusted). Items with misfit were excluded, as item misfit indicates the existence of multiple dimensions.

Local response dependency: Local response dependency is present, if the response to one item determines the response to another or two items depend on a further common variance source. Correlations above 0.30 in the correlation matrix identified this dependency and led to the exclusion of one item of the correlated pair of items.

Differential Item Functioning (DIF): DIF causes bias in measurements, reduces test fairness and can influence fit to the Rasch model. It occurs when subgroups (e.g. women and men) respond differently to an item, even though they have an identical underlying level of functional health [37]. The analyses of DIF were calculated for seven variables (gender, age, educational level, employment status, intensity of pain, subjective limitations due to CVD and cardiovascular diagnoses) and were performed by variance-based statistic. Uniform DIF was indicated by a significant main effect (p≤ 0.05) of the person factor (e.g. age), non-uniform DIF was indicated by a significant interaction effect (p≤ 0.05). Items with DIF were excluded.

Unidimensionality: The procedure proposed by Smith [38] was used to verify the unidimensionality of the final item bank. Therefore, two subsets of items were generated (positive vs. negative correlation between items and the first residual factor), which formed the basis of independent t-tests for each person. The number of significant tests should be less than 5% of the total number of tests.

Targeting of the scale (i.e. how well the items of the scale can appropriately target the patients being measured): The targeting of the scale was assessed by comparing the location of the items (fixed to zero logits) with the location of the participants in the person-item threshold distribution graph.

Reliability: The internal consistency reliability of the item bank was determined by the Person Separation Index (PSI). A PSI score of at least 0.85 for individual use (e.g. for a single patient) and at least 0.70 for group use (e.g. for research purpose) is regarded as sufficient (Table 1).

**Table 1:** Characteristics of the sample

	**N = 714**		**N = 368**	
	**N**	**%**	**N**	**%**
**Gender**				
Female	178	24.9	93	25.3
Male	537	75.1	275	74.7
**Age in years**				
≤50	150	20.9	83	22.6
51-60	307	42.7	148	40.2
≥61	262	36.4	137	37.2
**Family status**				
Single	46	6.5	27	7.5
In partnership	42	6.0	19	5.3
Married	508	72.2	256	71.1
Divorced/separated	65	9.2	37	10.3
Widowed	43	6.1	21	5.8
**Educational level**				
No graduation	1	0.1	1	0.3
Up to 9 years of school	306	44.3	157	43.9
Up to 10 years of school	201	29.1	111	31.0
Over 10 years of school	183	26.5	89	24.9
**Monthly income**				
<1000€	67	9.7	35	9.7
1001-2000€	210	30.4	114	31.9
2001-3000€	202	29.2	102	28.5
>3001€	212	30.7	107	29.9
**Employment status**				
Employed	429	60.8	216	58.7
Unemployed	277	39.2	152	41.3
**Intensity of pain**				
No pain	151	22.0	78	22.4
Moderate pain	424	61.9	207	59.5
Severe pain	110	16.1	63	18.1
**Subjective limitations due to CVD**				
No limitations	116	17.7	56	16.7
Mild limitations	243	37.0	116	34.6
Moderate limitations	240	36.6	135	40.3
Strong limitations	57	8.7	28	8.4
**Specific cardiovascular diagnoses**				
I10	87	12.1	45	12.2
I20-25	414	57.4	196	53.3
I30-52	107	14.9	61	16.6
I20-25 & I30-52	112	15.6	66	17.9
**Comorbid mental diseases**				
No	564	78.7	292	79.3
Yes	153	21.3	75	20.7
**Number of comorbid somatic diseases**				
0	100	14.0	48	13.1
1	226	31.5	109	29.7
2	193	27.0	106	28.9
3	136	19.0	69	18.8
≥4	61	8.5	35	9.5

## Results

### Participants

Most patients were male (75.1%), older than 50 years (79.1%), married (72.2%), employed (60.8%) and had at least 10 years of school attended (55.6%) (Table 1). More than half of the patients had ischemic heart disease (57.4%), followed by other forms of heart disease (14.9%), essential primary hypertension (12.1%), or a combination of ischemic heart disease and other forms of heart disease (15.6%). 86.0% of the participants had at least one comorbid somatic disease and 21.3% at least one comorbid mental disorder.

Owing to the block test design a subsample of patients answered an insufficient number of items to conduct the Rasch analysis. Therefore, 352 patients had to be excluded in course of the Rasch analysis. This led to a final calibration sample of 368 CVD patients. The two samples (N=352, N=368) did not differ significantly in socio-demographic and disease-specific variables.

### Results of confirmatory factor analysis

Eight items of the initial item pool (181 items) were excluded due to low factor loadings. The remaining 173 items had factor loadings of 0.50 to 0.91. RMSEA was lower than the required value of 0.10 (RMSEA=0.096), confirming that the relationship between the items can be assumed to be sufficiently determined by one single underlying factor.

### Results of Mokken analysis

22 items were excluded due to low values of Loevinger’s H. For the remaining item pool (151 items) Loevinger’s H was 0.63 confirming double monotonicity (0.50≤H: strong scale) (Table 2).

**Table 2:** Rasch parameters of the ADLib-cardio

**At the moment I’m able to accomplish the following activities without help…**		**Location (SE)**	**Residual (df)**	**Chi-square (probability)**	**Scoring structure**
FM057	Carrying a light object over 5 meters, e.g. a plate or a teapot	2.98 (0.19)	0.34 (194.92)	1.79 (0.62)	0 1 2 3 4
FS022	Putting on and taking off a coat or a jacket, including using the fastener	2.84 (0.21)	-1.21 (106.74)	4.06 (0.26)	0 1 2 3 4
FM111	Going up and down one single curbside or stair	2.72 (0.21)	-1.15 (107.67)	4.47 (0.22)	0 1 2 3 4
FM071	Removing the packaging of small objects	2.37 (0.19)	-0.50 (104.88)	1.54 (0.67)	0 1 2 3 4
FM068	Pushing and moving a light object with the feet, e.g. a ball	1.67 (0.25)	-1.01 (123.45)	5.02 (0.17)	0 1 2 2 2
FM001	Going to bed	1.49 (0.22)	-0.77 (125.3)	4.83 (0.18)	0 1 2 2 2
FM011	Getting up from a chair with the aid of the arms	0.56 (0.16)	0.20 (195.85)	0.32 (0.96)	0 1 2 2 2
FS014	Washing and drying my hair	0.54 (0.16)	-0.57 (194.92)	2.52 (0.47)	0 1 2 2 2
FM029	Sitting longer than 10 minutes	0.46 (0.17)	-0.24 (155.01)	2.26 (0.52)	0 1 2 2 2
FM008	Getting up from a sitting position, for example from a chair	0.41 (0.19)	-1.05 (109.53)	2.63 (0.45)	0 1 2 2 2
FM132	Travelling with public transportation more than half an hour	0.23 (0.19)	-1.10 (107.67)	3.17 (0.37)	0 1 2 2 2
FH042	Getting groceries for 1–2 days	0.18 (0.18)	1.35 (119.74)	0.86 (0.83)	0 1 2 2 2
FM093	Walking a short distance (up to 5 minutes)	0.12 (0.16)	-0.05 (153.15)	3.09 (0.38)	0 1 2 2 2
FM050	Pouring from a big pot	0.00 (0.14)	-1.31 (195.85)	2.43 (0.49)	0 1 2 2 2
FM016	Bending over to pick up a small object, e.g. a crumpled-up paper	-0.14 (0.15)	-0.14 (156.86)	2.51 (0.47)	0 1 2 2 2
FM133	Using public transportation, e.g. a bus or a train	-0.15 (0.16)	-1.43 (142.94)	4.25 (0.24)	0 1 2 2 2
FM087	Reaching behind the back to pull a belt through the loop	-0.23 (0.13)	-1.27 (195.85)	6.89 (0.08)	0 1 2 2 2
FH027	Taking bottles to the recycling container	-0.33 (0.16)	-0.32 (138.3)	5.94 (0.11)	0 1 2 2 2
FM128	Getting into or out of the car	-0.43 (0.13)	-0.55 (197.7)	1.16 (0.76)	0 1 2 2 2
FM033	Standing for 5–10 minutes without a break	-0.44 (0.12)	0.86 (149.44)	1.27 (0.74)	0 1 2 3 3
FM015	Picking up a light object, e.g. a garment, while sitting on a chair	-0.48 (0.15)	-0.39 (126.23)	3.01 (0.39)	0 1 2 2 2
FH016	Making the bed	-0.54 (0.17)	-2.14 (105.81)	5.95 (0.11)	0 1 2 2 2
FM113	Going down a staircase over three floors	-0.82 (0.14)	-0.05 (109.53)	0.76 (0.86)	0 1 2 3 3
FS034	Pursuing normal leisure activities, e.g. cycling or going for a walk	-0.87 (0.13)	1.34 (105.81)	1.02 (0.80)	0 1 2 3 3
FM105	Walking on an uneven, rocky path	-0.96 (0.13)	0.06 (124.38)	0.84 (0.84)	0 1 2 3 3
FH051	Helping others, when they need my assistance	-0.97 (0.10)	1.27 (185.64)	6.18 (0.10)	0 1 2 3 3
FM076	Pushing open a heavy door	-0.99 (0.12)	0.76 (192.13)	6.16 (0.10)	0 1 2 2 2
FM108	Getting up 3–5 stairs (without a handrail)	-0.99 (0.13)	-0.31 (155.93)	0.73 (0.87)	0 1 2 2 2
FH020	Cleaning the floor	-1.12 (0.11)	-1.24 (172.64)	2.65 (0.45)	0 1 2 3 3
FM098	Walking for one hour on even pathways, e.g. on the pavement	-1.54 (0.16)	0.26 (104.88)	2.67 (0.44)	0 1 2 2 2
FM014	Getting up from the floor, for example from a kneeling position like after falling	-1.57 (0.10)	-0.31 (192.13)	3.96 (0.27)	0 1 2 3 3
FM106	Going on longer hikes on uneven paths, e.g. in the forest or on field roads	-1.96 (0.09)	0.54 (186.56)	5.88 (0.12)	0 1 2 3 4
FM104	Walking on a rising path	-2.04 (0.10)	0.62 (199.56)	4.61 (0.20)	0 1 2 3 3

### Results of Rasch analysis

97 items were excluded due to individual item misfit or local response dependency, based on a step by step process. In a first step, items showing local dependency were deleted (76 items). Then those items were deleted, which led to higher PSI and fit residual values of persons and items (21 items). 21 items showed DIF with regard to the different levels of the variables “gender” (15 items), “age” (5 items), “pain” (1 item) and were therefore excluded. 28 of the 33 items of the final item bank had to be rescored (Table 2).

Data of the final item bank fitted to the Rasch model with a nonsignificant probability value of the item-trait interaction score (chi-square=105.42, df=99; p=0.31). Statistics of the residuals for items (mean=-0.29, SD=0.87) and for persons (mean=-0.30, SD=0.96) were close to perfect values (mean=0.00, SD=1.00) and supported model fit. Items fit residuals values varied between -2.14 and 1.35 and thus remained within the uncritical ±2.50 range. Item chi-square values varied between 0.32 and 6.89 and all probability values were higher than the Bonferroni adjusted alpha value.

**Figure 1:**
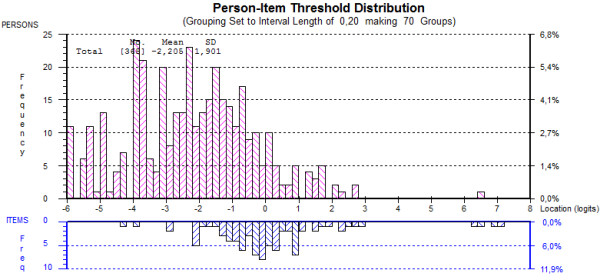
Person-item threshold distribution of the ADLib-cardio.

All 33 items of the final item bank were free from DIF. The local response independency of the items was confirmed by the correlation matrix of the item bank with no correlations above 0.30. The results of paired t-tests supported the unidimensionality of the item bank with only 4.23% of t-tests showing a significant difference Figure 1.

The category threshold parameters of the item bank covered a range of 7.19 logits (-4.32 to 2.87) and could thus capture a wide spectrum of ADL (Figure 1). The easiest item in the bank was item FM057 “Carrying a light object over 5 meters, e.g. a plate or a teapot”. The most difficult item was FM104 “Walking on a rising path”. Location of the items varied between 2.98 and -2.04, with a mean of 0.00 (SD=1.33). The mean person location was -2.21 (SD 1.33), indicating that the sample showed a higher level of ADL than the average level of ADL by the item bank.

The Person Separation Index had a score of 0.81. This demonstrated good person separation reliability for group use (PSI≥0.70).

## Discussion

The Activities of Daily Living item bank for cardiovascular patients (ADLib-cardio) demonstrates good psychometric properties, covers a wide spectrum of ADL, shows comprehensive test fairness and measures ADL unidimensionally. The 33 items of the item bank cover both basic (e.g. going to bed) and instrumental (e.g. helping others, when they need my assistance) aspects of ADL, thus covering a broad spectrum of patients´ functional status. Compared with other generic ADL item banks such as the ALDS item bank [18] and the PROMIS physical functioning item bank [19], the ADLib-cardio is free of DIF with regard to cardiovascular diagnoses. Moreover, the ADLib-cardio is free of DIF with regard to six further socio-demographic (gender, age, educational level, employment status) and medical variables (intensity of pain, subjective limitations due to CVD). The test-fairness of the ADLib-cardio is of particular importance, as an unfair item can heavily impact results of instruments with a low item number [39].

The ADLib-cardio is a psychometrically sound assessment instrument with 33 items. However, as ADL is only one dimension of a comprehensive psycho-social assessment of cardiovascular patients, it seems important to further improve the test duration regarding both patients´ time needed to complete the test and diagnosticians´ time needed to evaluate test results. Thus, the main advantage of the ADLib-cardio is its quality to provide the basis for the development of both CAT and short form questionnaires. It is possible to create a short form questionnaire for basic and instrumental ADL or a test with parallel versions for pre-post measurement [40,41]. The development of the ADL-CAT-cardio would provide an economic possibility to assess ADL in cardiovascular patients. In case of different instruments developed on the basis of the ADLib-cardio, results would remain comparable across all tests [20].

All ADLib-cardio-based instruments can be used both for diagnostic and evaluative purposes. Diagnostically they can identify patients with a critical level of functional health and determine the severity level of ADL. A recent study showed that the presence of ADL limitations is the best predictor of further functional decline [1]. Thus, patients´ ADL level might help to determine when intensified efforts to prevent further functional decline are indicated. Concerning evaluation, they can make even small changes (e.g. to treatment) objectively measurable [14,20]. This is of particular importance for monitoring the progress of patients´ functional health status as one of the most important predictors of morbidity and mortality next to depression [42-44]. The ADLib-cardio, as an instrument sensitive to change, might also help to further examine the bi-directional relationship between ADL and depression. Depression is frequent in CVD patients [45-47] and associated with a decreased health status [48,49] and increased cost [50]. Thus, mapping the processes that impact both functional status and depression following cardiac events might help to further improve health care in CVD patients.

The present study shows some limitations. First, we had to exclude a large number of items. This might have been partly due to the fact that the content of the item bank was not informed initially by patient input but by extant questionnaires, which might have contributed to the high level of item misfit. To lower the number of excluded items, it would have been possible to use otherwise good items that exhibit DIF by explicitly modeling the DIF (i.e. using different parameters for the same item for different groups). For example, items displaying DIF for gender could be administered to males or females only, or have different scoring parameters for each group. Similarly, items showing local dependency could be kept in an item bank, if ‘testlet” or “multi-stage” adaptive designs are used, where items are administered in blocks, adapting only between blocks [51]. However, as the item bank with 33 items is still sufficiently large for developing assessment instruments such as Computer adaptive tests, we decided to omit items with DIF and local dependency in favor of a consistent and easy-to-use item bank for all CVD rehabilitation patients. Second, the sample had to be reduced in size due to the large exclusion of items (181 to 33 items) and the chosen block test design for data collection. This limitation, however, seems to be negligible, as the remaining sample did not differ meaningfully from the overall sample. Moreover, the sample size was still sufficiently large for calibrating the item pool. Third, while the ADLib-cardio covers a wide spectrum of ADL, its potential for exact measurement of a very restricted or unrestricted level of ADL is limited. Thus, constructing and analyzing additional items for these areas might further improve the ADLib-cardio. Fourth, given that the response categories of 28 items had to be merged, the response scale (1 to 5) might have been too differentiated in light of the patients´ ability to discriminate between categories. It is still possible to answer the items of the ADLib-cardio using the homogeneous five-point response format and thereby keep the answering simple and economic. However, the categories need to be recoded afterwards either manually or automatically as part of the CAT according to the scaling structure. Finally, while a response rate of 35% is not unusual for this type of studies it might still impact the representativeness of the sample. However, given that this study aims to assess the psychometric properties of a patient reported outcome assessment tool for patients with cardiovascular diseases, the representativeness of the sample is not as important as it is for other types of studies (e.g. population-based studies) as generalizability is not the aim here.

## Conclusions

The calibrated, unidimensional item bank covers a wide spectrum of ADL and can be used to improve the recognition of disability in ADL in cardiovascular health care. The ADLib-cardio shows good psychometric properties and provides the basis for a CAT and for short form screening questionnaires in rehabilitation patients with CVD. Thereby, the comprehensive developing process and the ambitious statistical procedure indicate a high validity of the ADLib-cardio. Assessment instruments derived from the ADLib-cardio such as CAT, however, need to further examine the convergent and discriminant validity of the respective tests [52]. With regard to the development of the ADL-CAT-cardio, simulation studies (e.g. with the free software “Firestar” [53]) and clinical practice tests are needed to determine the efficiency (e.g. item number; test time) of the ADLib-cardio as the basis for a CAT. Finally, a calibration in cardiovascular populations other than inpatient rehabilitation patients (e.g. outpatients or acute inpatients, people with CVD from the general population) would further increase generalizability of the ADLib-cardio. Additional research could also show whether the ADLib-cardio is transferable to other populations than CVD patients, such as to patients with other diseases.

## Competing interests

The authors declare that they have no competing interests.

## Authors’ contributions

HB was responsible for the study design and wrote the manuscript together with BA and AH. BA and AH collected the data and performed the statistical analyses. JB, MB and MW participated in study design, manuscript writing, editing and reviewing. All authors read and approved the final version of the manuscript.
